# Conservation of the “Outside-in” Germination Pathway in *Paraclostridium bifermentans*

**DOI:** 10.3389/fmicb.2018.02487

**Published:** 2018-10-17

**Authors:** Disha Bhattacharjee, Joseph A. Sorg

**Affiliations:** Department of Biology, Texas A&M University, College Station, TX, United States

**Keywords:** spore, germination, Clostridium, DPA, cortex

## Abstract

*Clostridium difficile* spore germination is initiated in response to certain bile acids and amino acids (e.g., glycine). Though the amino acid-recognizing germinant receptor is unknown, the bile acid germinant receptor is the germination-specific, subtilisin-like pseudoprotease, CspC. In *C. difficile* the CspB, CspA, and CspC proteins are involved in spore germination. Of these, only CspB is predicted to have catalytic activity because the residues important for catalysis are mutated in the *cspA* and *cspC* sequence. The CspB, CspA, and CspC proteins are likely localized to the outer layers of the spore (e.g., the cortex or the coat layers) and not the inner membrane where the Ger-type germinant receptors are located. In *C. difficile*, germination proceeds in an “outside-in” direction, instead of the “‘inside-out” direction observed during the germination of *Bacillus subtilis* spores. During *C. difficile* spore germination, cortex fragments are released prior to the release of 2,4-dipicolinic acid (DPA) from the spore core. This is opposite to what occurs during *B. subtilis* spore germination. To understand if the mechanism *C. difficile* spore germination is unique or if spores from other organisms germinate in a similar fashion, we analyzed the germination of *Paraclostridium bifermentans* spores. We find that *P. bifermentans* spores release cortex fragments prior to DPA during germination and the DPA release from the *P. bifermentans* spore core can be blocked by high concentrations of osmolytes. Moreover, we find that *P. bifermentans* spores do not respond to steroid-like compounds (unlike the related *C. difficile* and *P. sordellii* organisms), indicating that the mere presence of the Csp proteins does permit germination in response to steroid compounds. Our findings indicate that the “outside in” mechanism of spore germination observed in *C. difficile* can be found in other bacteria suggesting that this mechanism is a novel pathway for endospore germination.

## Introduction

The endospore-forming *Paraclostridium bifermentans* belongs to the Clostridia family and *P. bifermentans* subsp. *malaysia* is the only known anaerobic larvicidal toxin producer whose toxins target *Anopheles* and *Aedes* mosquitoes (Qureshi et al., 2014). Due to the anaerobic nature of *P. bifermentans* vegetative cells, the organism likely survives between hosts in the form of a dormant spore. Endospore formation is conserved in many Bacilli and Clostridia, thoughthe sporulation pathway exhibits some differences between organisms (Fimlaid et al., 2013). Despite these differences, the overall architecture of the metabolically dormant spore is conserved.

Located in the center of the spore, the core contains DNA, RNA, ribosomes, and protein, and the core has a low water content with high amounts of 2,4-dipicolinic acid (DPA), which provides resistance against heat (Permpoonpattana et al., 2011; Setlow, 2014). Surrounding the spore core is a thin germ cell wall, which becomes the cell wall peptidoglycan of the vegetative cell upon germination (Setlow, 2014), and a thick cortex peptidoglycan layer composed of N-acetylglucosamine (NAG), N-acetylmuramic acid (NAM), and muramic-δ-lactam residues. Finally, layers of coat proteins surround the cortex layer and protect the spore from environmental insults.

During spore development, receptors that identify suitable environmental conditions for metabolism and growth are incorporated into the spores by the mother cell or the forespore (Errington, 2003; Francis et al., 2013; Bhattacharjee et al., 2016b). Upon binding to small molecule germinants, these receptors trigger the irreversible germination process (Bhattacharjee et al., 2016b). The germination process has been described best in the model spore-forming bacterium, *Bacillus subtilis*. *B. subtilis* spores germinate in response to L-alanine or a mixture of L-asparagine, glucose, fructose and potassium ions (AGFK) (Setlow, 2014). These germinants are thought to interact with their respective germinant receptors embedded within the inner spore membrane (Moir et al., 2002; Setlow, 2014). Whereas the GerBA-BB-BC and GerKA-KB-KC germinant receptor responds to AGFK, the GerAA-AB-AC germinant receptor responds to L-alanine (Setlow, 2014). Though the signals that activate spore germination in other organisms vary, nearly all endospore-forming organisms, studied to date, encode orthologs of the transmembrane Ger*-*type germinant receptor (Paredes-Sabja et al., 2011). Germinant receptor activation leads to the release of monovalent cations and the large depot of DPA, from the channel composed of the SpoVA proteins, resulting in rehydration of the spore core (Setlow, 2014). Subsequently, two redundant spore cortex lytic enzymes (SCLEs), CwlJ and SleB, are activated and, through their combined actions, the cortex is degraded (Setlow, 2014). Cortex degradation allows for full core rehydration, loss of dormancy, restoration of metabolism and, finally, outgrowth of a vegetative cell from the germinated spore.

Unlike what is found in *B. subtilis* and other spore-forming bacteria, *Clostridium difficile* [also *Clostridioides difficile* (Lawson et al., 2016; Oren and Garrity, 2016)] does not encode orthologs of the known *ger-*type germinant receptors, suggesting that *C. difficile* spore germination occurs through a novel mechanism or uses novel signals (Sebaihia et al., 2006). *C. difficile* initiates germination in response to cholic acid derivatives (bile acids) and amino acids (e.g., glycine or alanine), while chenodeoxycholic acid derivatives are competitive inhibitors of cholic acid-mediated germination (Sorg and Sonenshein, 2008, 2009, 2010; Paredes-Sabja et al., 2014; Bhattacharjee et al., 2016b). Though necessary for *C. difficile* spore germination, bile acids are not sufficient (Sorg and Sonenshein, 2008). A second, amino acid-based signal is required to activate the germination process. Glycine is the best co-germinant, but most other amino acids can substitutes with varying efficiencies (Sorg and Sonenshein, 2008; Shrestha et al., 2017; Shrestha and Sorg, 2018). Recently, calcium was reporting to function as an enhancer of *C. difficile* spore germination, however, it is unclear if it acting in the role of a *bona fide* germinant or if it functions as a co-factor/essential component of germination proteins (Kochan et al., 2017). Previously, our lab determined that the bile acid germinant receptor is the germination-specific, subtilisin-like, pseudoprotease, CspC (Francis et al., 2013; Paredes-Sabja et al., 2014; Bhattacharjee et al., 2016b). The Csp proteases were originally studied in *Clostridium perfringens* (Shimamoto et al., 2001; Masayama et al., 2006; Paredes-Sabja et al., 2011). In *C. perfringens*, the CspA, CspB, and CspC proteases cleave the inactive SCLE, pro-SleC, to its active form resulting in cortex degradation (Shimamoto et al., 2001). The *C. perfringens* CspB, CspA, and CspC proteins can be extracted from spore coats (Shimamoto et al., 2001). But, in the article that describes this, no control was given for cortex-localized proteins suggesting that these proteins could be coat- or cortex-localized (Shimamoto et al., 2001). In *C. difficile*, the *cspB* and *cspA* sequences are fused, translationally, and *cspC* is encoded downstream of *cspBA* (Adams et al., 2013). Though CspB is produced as a fusion with CspA, the timing for interdomain cleavage and the fate of CspA after cleavage is still unknown. However, the loss of CspA leads to a significant decrease in spore germination, and CspA has been shown to control CspC levels in the spore (Francis et al., 2013; Kevorkian et al., 2016). CspB is capable of processing pro-SleC to its active form. However, the catalytic triads that are characteristic of subtilisin-like proteases are absent in both CspA and CspC, and loss of *cspA* or *cspC* negatively affects spore germination, suggesting that CspA and CspC function in a regulatory role and not a catalytic role (Adams et al., 2013; Francis et al., 2013).

Based upon the predicted location of CspB, CspA, CspC, and SleC (near the cortex layer and not in/on the inner spore membrane) (Miyata et al., 1997), we hypothesized that *C. difficile* spore germination may be initiated differently than what had been described in other endospore-forming bacteria (Francis et al., 2015). Specifically, we hypothesized that bile acids and glycine would stimulate cortex degradation prior to the release of DPA from the spore core (a process opposite to what is observed during *B. subtilis* spore germination). Indeed, cortex degradation precedes release of DPA from the spore core and the release of DPA is dependent on the osmotic changes that occur at the inner membrane when cortex is degraded (Francis et al., 2015; Francis and Sorg, 2016). This suggests that *C. difficile* spore germination proceeds through a novel spore germination pathway where the germinants stimulate cortex degradation. In order to understand if this mechanism of germination is unique to *C. difficile* or if other organisms share this pathway of spore germination, we analyzed germination in *P. bifermentans*. *P. bifermentans* encodes a *csp* locus that is similar to what is observed in *C. difficile* where the *cspB* and *cspA* sequences are translationally fused and *cspA* and *cspC* do not encode proteins with complete catalytic triads. Moreover, *P. bifermentans* encodes a peptidoglycan binding protein that is 56% similar to the peptidoglycan degrading protein SleC from *C. difficile*. Herein, we find that *P. bifermentans* cortex degradation precedes the release of DPA from the spore core and the release of DPA can be delayed by high concentrations of osmolytes. Our data suggest that, like *C. difficile*, *P. bifermentans* spores germinate through an “outside-in” mechanism and add to the list of organisms that germinate through this novel pathway of spore germination.

## Results

### Identifying Potential *P. bifermentans* Germination Receptors

**FIGURE 1:**
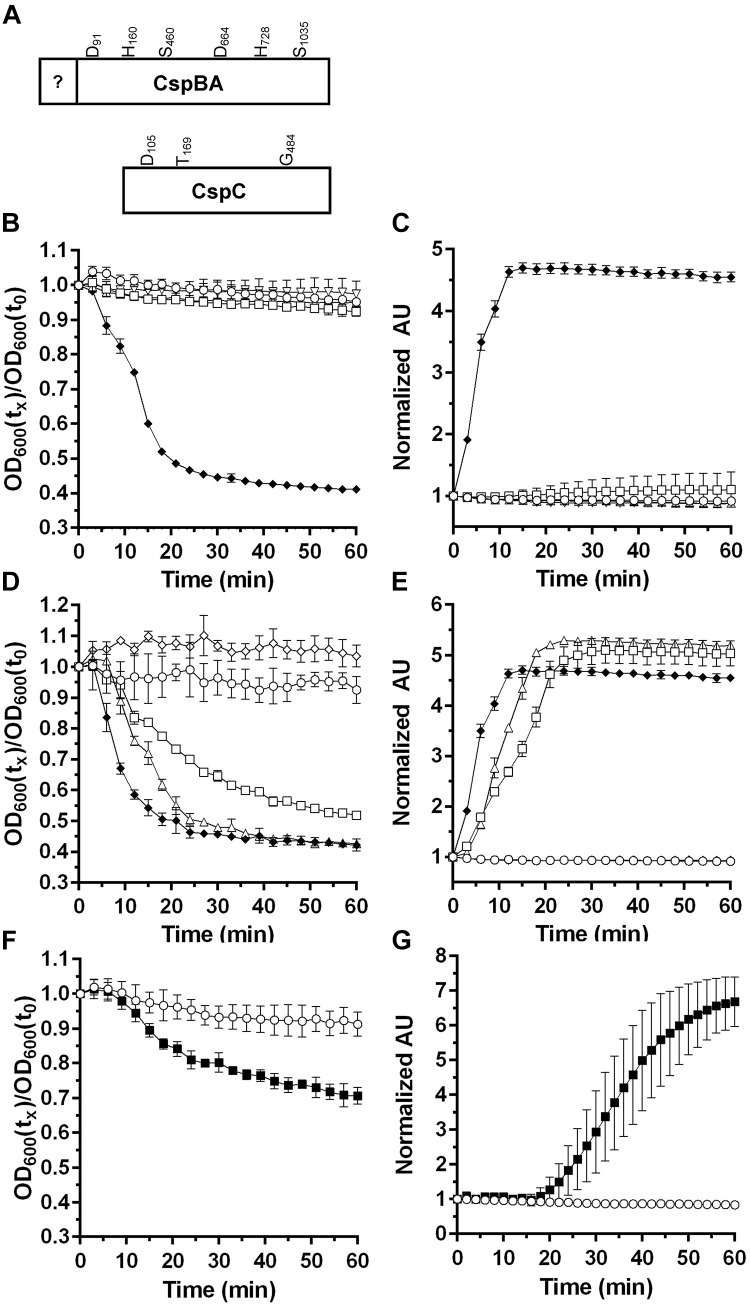
Characterizing the germinants of *P. bifermentans* spores. **(A)** Illustration of the *P. bifermentans* CspBA and CspC proteins and the location of the would-be catalytic residues. Germination of *P. bifermentans* spores was analyzed by changes in OD_600_ **(B,D,F)** and Tb^3+^ fluorescence upon complexing with DPA **(C,E,G)**. Germination assays were conducted in 50 mM HEPES, 100 mM NaCl at pH 7.5 buffer supplemented amino acids. **(B,C)** Spores alone, 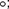 50 mM L-alanine 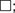 50 mM L-arginine, Δ; 50 mM L-phenylalanine, open inverted triangle; 50 mM L-alanine, 5 mM L-phenylalanine, 5 mM L-arginine, 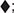 **(D,E)**. Spores alone, 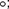 50 mM L-alanine, 5 mM L-arginine, 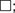 50 mM L-alanine, 5 mM L-phenylalanine, Δ; 5 mM L-phenylalanine, 5 mM L-arginine, 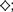 50 mM L-alanine, 5 mM L-phenylalanine, 5 mM L-arginine, 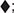 **(F,G)** Spores alone, 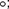 50 mM L-alanine, 5 mM L-leucine, 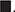 Data points represent the average from three independent experiments and error bars represent the standard deviation from the mean.

Prior research from our laboratory demonstrated that there are differences between mechanisms for the initiation of spore germination observed in *B. subtilis* and *C. difficile* (Francis et al., 2015; Francis and Sorg, 2016). The primary difference is the presence of *csp*-type germinant receptors and the absence of *ger-*type receptors in *C. difficile* (Paredes-Sabja et al., 2011; Francis et al., 2013). *P. bifermentans* also encodes homologs to *C. difficile* *cspBA* and *cspC* (**Figure 1A**). In the unannotated *P. bifermentans* whole genome sequence, the *cspBA* gene encodes a truncated protein. Upon sequencing the *P. bifermentans* *cspBA* gene, we found that there is a sequencing error in the *cspBA* gene in the deposited NCBI sequence in the form of a deletion of an adenine within a stretch of consecutive adenine residues (**Supplementary Figure S1**). This indicates that the *P. bifermentans* CspBA protein sequence is intact and has the potential to function similarly to what is observed in *C. difficile*.

To determine if *P. bifermentans* encodes a *gerAA* ortholog, we used BLAST to search the *P. bifermentans* genome for the *B. subtilis* GerAA protein. Interestingly there is an annotated *gerA* sequence in *P. bifermentans*. Using this protein sequence as a query, we found that the *P. bifermentans* sequence most-closely matches the *B. subtilis* SpoVAF spore protein and not GerA. Taken together, these results suggest that *P. bifermentans* does not encode *ger*-type germinant receptors but encodes a *csp* locus that is similar to that of *C. difficile*.

### Germination of *P. bifermentans* Spores in Response to Amino Acids

Previously, the germinants for *P. bifermentans* spores were identified (Gibbs, 1964; Waites and Wyatt, 1971). In order to dissect the mechanism of *P. bifermentans* spore germination, we monitored germination using both change in OD_600_ nm (which measures the sum of events during loss of dormancy) and release of DPA (as measured by Tb^3+^ fluorescence). Purified spores were suspended in HEPES-buffer alone or supplemented with L-alanine (A), L-arginine (R), L-phenylalanine (F), or all three amino acids (ARF). *P. bifermentans* spores rapidly germinated in the presence of ARF but not when exposed to the amino acids individually (**Figure 1B**). When DPA release was assayed, ARF stimulated the rapid release of DPA from the germinating *P. bifermentans* spores (**Figure 1C**). Next, we tested whether binary combinations of the amino acids could stimulate *P. bifermentans* spore germination. Though ARF was the best activator of spore germination, AF and AR could also stimulate germination as measured by OD change (**Figure 1D**) and DPA release (**Figure 1E**). We then tested other amino acids to understand if other amino acids can synergize with L-alanine to stimulate *P. bifermentans* spore germination. In doing so, we found that L-leucine can function as a germinant with L-alanine as measured by both germination at OD_600_ (**Figure 1F**) and DPA release (**Figure 1G**). As determined by the rate of OD change and the rate of DPA release, AF was a weaker activator of spore germination than ARF, AR was weaker than AF and AL was weaker than AR (ARF > AF > AR > AL). These results suggest that *P. bifermentans* spore germination requires at least L-alanine and another amino acid (L-arginine, L-phenylalanine, or L-leucine) and that L-alanine is an essential, but not sufficient, germinant for *P. bifermentans* spores.

### Bile Acids Do Not Influence *P. bifermentans* Spore Germination

**FIGURE 2:**
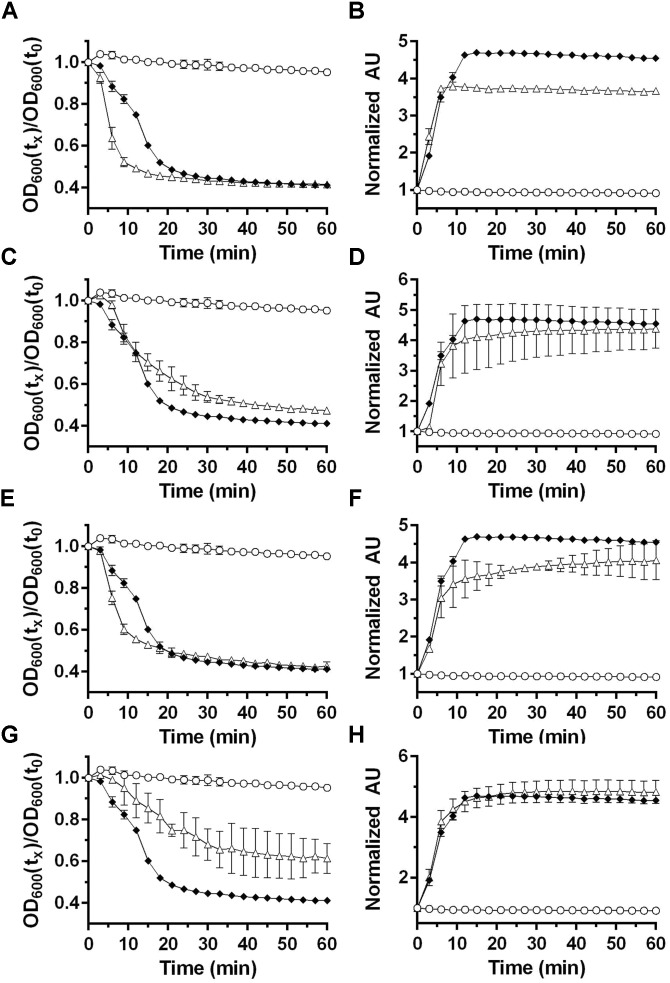
Analyzing the influence of bile acids on *P. bifermentans* spore germination. Germination of *P. bifermentans* spores was analyzed by changes in OD_600_ **(A,C,E,G)** and Tb^3+^ fluorescence upon complexing with DPA **(B,D,F,H)**. Spores, 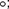 50 mM L-alanine, 5 mM L-phenylalanine, 5 mM L-arginine, 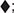 50 mM L-alanine, 5 mM L-phenylalanine, 5 mM L-arginine, indicated compound, Δ. **(A,B)** 2 mM taurocholic acid; **(C,D)** 2 mM chenodeoxycholic acid; **(E,F)** 2 mM deoxycholic acid; **(G,H)** 2 mM triton X-100. Data points represent the average from three independent experiments and error bars represent the standard deviation from the mean.

*Clostridium difficile* germination is activated in response to a combination of cholic acid-class bile acids and an amino acid (e.g., glycine) (Sorg and Sonenshein, 2008; Howerton et al., 2011; Bhattacharjee et al., 2016b). In *Clostridium sordellii*, a related organism, ARF-mediated spore germination is enhanced by steroid-like compounds, including bile acids, and *C. sordellii* encodes orthologs of *C. difficile* *cspBA* and *cspC* (Liggins et al., 2011). Therefore, we hypothesize that steroid/bile acid recognition may be a property of organisms that encode the *cspBAC* locus. To determine whether bile acids can be recognized in *P. bifermentans*, we germinated *P. bifermentans* spores in presence of various bile acids [taurocholic acid (TA), deoxycholic acid (DCA), and chenodeoxycholic acid (CDCA)] (**Figure 2**). *P. bifermentans* spores did not germinate in response to bile acids alone and still required ARF to activate spore germination. Interestingly, the OD change during *P. bifermentans* spore germination was faster in presence of 2 mM TA (**Figure 2A**), similar to what was observed in *C. sordellii*, though the DPA release by *P. bifermentans* spores was unaffected (**Figure 2B**). CDCA, normally an inhibitor of germination in *C. difficile*, did not affect germination by *P. bifermentans* spores (**Figures 2C,D**). However, similar to TA, DCA increased the rate of germination measured by OD (**Figure 2E**) but did not influence the release of DPA (**Figure 2F**). To confirm that these observations are not due to a detergent-like effect of TA or DCA, spores were germinated in presence of 2 mM Triton X-100. Triton X-100 did not increase the rate of germination by *P. bifermentans* spores either by OD (**Figure 2G**) or DPA release (**Figure 2H**). These results suggest that, though TA and DCA increase the rate of OD change during germination, DPA release is unaffected by bile acids suggesting that they do not influence *P. bifermentans* spore germination and the observed effects on OD are likely an artifact.

### Cortex Degradation Precedes DPA Release During *P. bifermentans* Spore Germination

**FIGURE 3:**
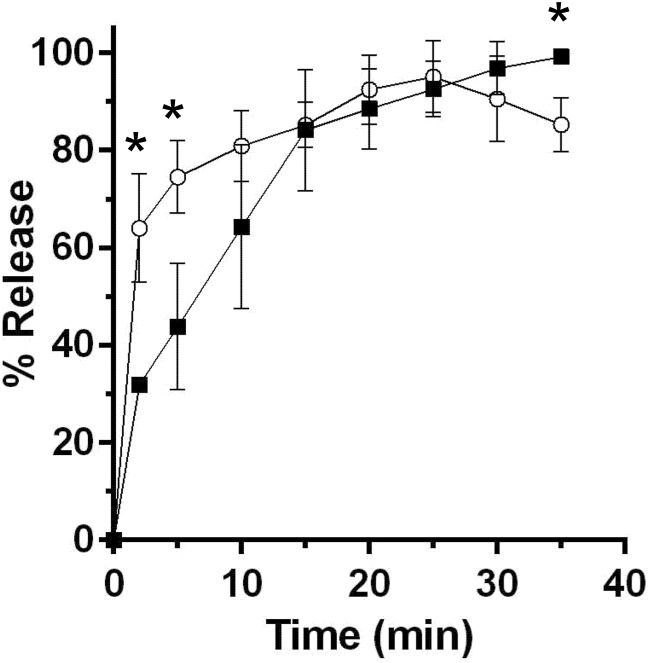
Cortex degradation precedes DPA release during *P. bifermentans* spore germination. *P. bifermentans* spores were germinated in the presence of L-alanine, L-arginine, and L-phenylalanine. At the indicated times, samples were taken to analyze cortex fragment release 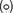 and for DPA release 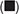. Data points represent the average from three independent experiments and error bars represent the standard deviation from the mean. Statistical significant was determined using a two-way ANOVA with Sidak’s multiple comparisons test. The asterisk marked points indicate statistical significance (*p*-value < 0.05).

A major difference between *C. difficile* and *B. subtilis* spore germination is the timing/order of the release of DPA and cortex fragments (Francis et al., 2015). To understand if *P. bifermentans* spores release cortex fragments before DPA (similar to *C. difficile* spore germination) or vice versa (similar to *B. subtilis* spore germination), we utilized an assay, that detects the presence of reducing sugars during germination, as described previously (Francis et al., 2015). Spores were suspended in germination buffer supplemented with 100 mM ARF (the concentration of ARF was increased to 100 mM each in order to achieve higher levels of spore germination in this assay) and germination was monitored over time. At the indicated time points, samples were removed and processed for the presence of reducing sugars and the presence of DPA. Within 2 min after the induction of germination, we observed a statistically significant difference between the amount of released reducing sugar and DPA (**Figure 3**). This difference was also present at 5 min. However, by 10 min post-germinant addition, the fraction of released cortex fragments and DPA were indistinguishable. Because we observed the presence of reducing sugars in the germination medium before we observed the presence of DPA, these results suggest that cortex degradation occurs prior to DPA release during *P. bifermentans* spore germination.

### Analyzing DPA Release in the Presence of High Concentrations of Osmolytes

**FIGURE 4:**
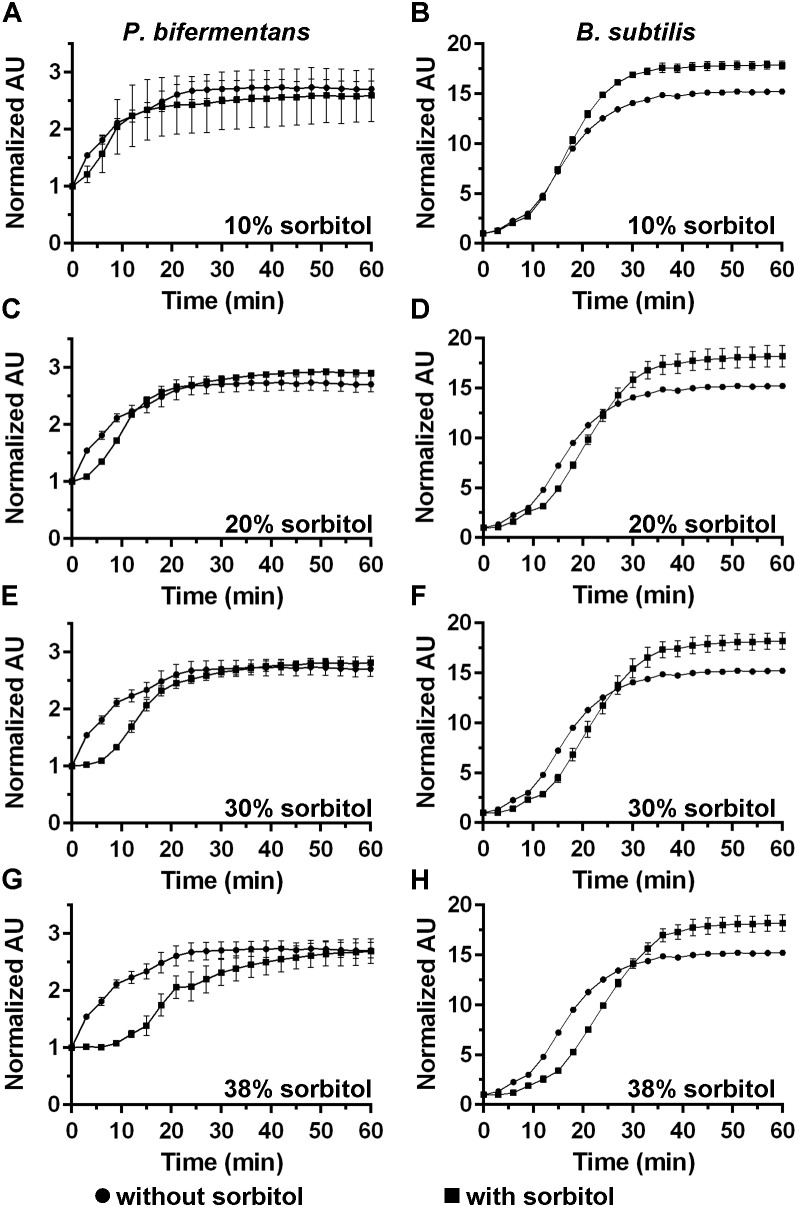
Analyzing DPA release in the presence of high concentrations of sorbitol. DPA release from germinating *P. bifermentans* spores **(A,C,E,G)** or from germinating *B. subtilis* spores **(B,D,F,H)** was analyzed germinated in presence of 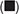 and in absence of 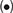 sorbitol. **(A,B)** 10% sorbitol; **(C,D)** 20% sorbitol; **(E,F)** 30% sorbitol; **(G,H)** 38% sorbitol. Data points represent the average from three independent experiments and error bars represent the standard deviation from the mean.

DPA release by germinating *C. difficile* spores can be delayed by high osmolyte concentrations (e.g., sorbitol) (Francis and Sorg, 2016). We hypothesized that if cortex degradation precedes DPA release during *P. bifermentans* spore germination, the release of DPA may be dependent on the osmotic changes that occur at the inner spore membrane. To test this hypothesis, we added increasing amounts of sorbitol to the germination buffer and monitored DPA release during germination by both *P. bifermentans* spores and *B. subtilis* spores (**Figure 4**). We could not simultaneously measure cortex degradation and DPA release during *P. bifermentans* spore germination. Due to unknown reasons, the ARF amino acids in the sorbitol-containing germination buffer reacted with the components of the cortex hydrolysis assay and did not yield a colorimetric signal. When *P. bifermentans* spores were suspended in germination buffer supplemented with 10% sorbitol (**Figure 4A**), we observed a short, but non-significant, delay in the release of DPA compared to spores suspended in germination buffer alone. There also was no significant delay in DPA release for germinating *B. subtilis* spores in 10% sorbitol (though sorbitol increased the total signal during *B. subtilis* spore germination; **Figure 4B**). When the amount of sorbitol was increased to 20% (**Figure 4C**), 30% (**Figure 4E**), and 38% (**Figure 4G**), the delay in DPA release by germinating *P. bifermentans* spores increased by nearly 15 min (**Figure 4G**). For *B. subtilis* spore germination, the presence of increasing concentrations of sorbitol slightly delayed DPA release but did not appear to be dose-dependent as observed for *P. bifermentans* (**Figures 4B,D,F,H**).

### Quantifying the Effects of Sorbitol on Spore Germination

**FIGURE 5:**
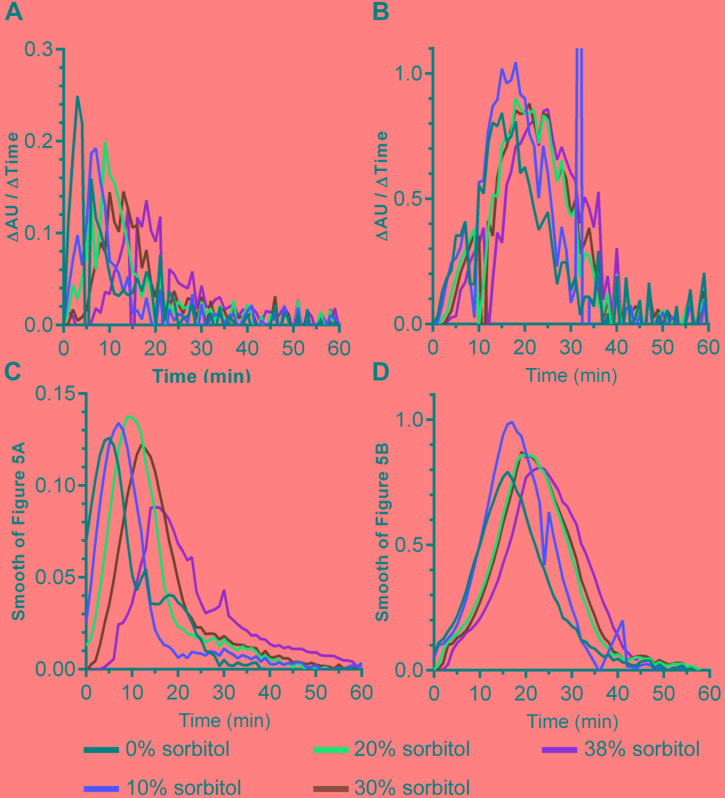
Quantifying the effects of sorbitol on spore germination. The maximum rates of spore germination in the presence or absence of sorbitol was determined by applying a first order derivative to the germination curves found in **Figure 4** for both *P. bifermentans* **(A,C)** and *B. subtilis* **(B,D)**. The raw data from the first order derivative **(A,B)** was smoothed using a rolling average of 8 surrounding data points for every data point in the plot **(C,D)**. Experiments were performed in triplicate and the plots are a representative of one of the replicates. The average maximum rate of DPA release from the triplicate samples are tabulated in **Table 1**.

The data in **Figure 4** suggest that *P. bifermentans* spores may be more susceptible to a sorbitol-mediated delay in DPA release than *B. subtilis* spores. A 2-way ANOVA analysis of each curve found that a significant delay in the initial DPA release can be observed during *P. bifermentans* and *B. subtilis* spore germination. To quantify this effect we determined the time at which the maximum rate of DPA release occurred by taking the first order derivative of the germination plots used to derive the data in **Figure 4**, **5**. Plotted in **Figures 5A,B** are the raw data from the derivative and show that *P. bifermentans* spores delay DPA release, in a dose dependent manner, upon sorbitol addition (**Figure 5A**). However, *B. subtilis* spores appear to be not as influenced as *P. bifermentans* spores (**Figure 5B**). To provide a clearer understanding of what is occurring in **Figures 5A,B**, we took a rolling average of the surrounding 8 data points for every data point in 5A and 5B to smooth the plots (**Figures 5C,D**). As shown in **Figure 5C** and quantified in **Table 1**, the time at which the maximum rate of DPA release occurs during *P. bifermentans* spore germination is delayed in a step-wise manner with increasing concentrations of sorbitol. However, this same step-wise delay is not nearly as dramatic during *B. subtilis* spore germination (**Figure 5D** and **Table 1**). These results suggest that: (i) high osmolyte concentrations prevent the release of DPA during *P. bifermentans* spore germination similar to prior observations during *C. difficile* spore germination; (ii) that germination by *P. bifermentans* spores occurs more similar to *C. difficile* than *B. subtilis*; and (iii) that, though mechanosensing may play a role during *B. subtilis* spore germination (Velasquez et al., 2014), other factors are influencing DPA release by *B. subtilis* spores (e.g., germinant receptors or GerD).

**Table 1:** Quantifying the sorbitol-dependent delay in DPA release (min).

	0% Sorbitol	10% Sorbitol	20% Sorbitol	30% Sorbitol	38% Sorbitol
*P. bifermentans*	5 ± 0.6	7.3 ± 0.3 (2.3)	9 ± 0 (4.0)	12.3 ± 0.3 (7.3)	17.7 ± 2.7 (12.7)
*B. subtilis*	16 ± 0	16.7 ± 0.3 (0.7)	20 ± 0.6 (4.0)	19.7 ± 0.7 (3.7)	22.3 ± 0.7 (6.3)


*The time at which the maximum rate of DPA release under the conditions in **Figures 5C,D** (smoothed data) occurred was tabulated. The values reported are the averages from three independent experiments ± SEM. Values in parentheses highlight the delay and are tabulated by subtracting the value at 0% sorbitol from the other values.*

## Discussion

Most spore-forming bacteria studied to date germinate using a mechanism similar to what has been described for *B. subtilis*. In *B. subtilis*, germinant recognition by the Ger-type germinant receptor results in the release of DPA from the core, likely through a channel composed of the SpoVA proteins. This event triggers the degradation of the spore cortex layer and the irreversible loss of dormancy. *C. difficile* spore germination is triggered by the combinatorial actions of certain bile acids and glycine (Sorg and Sonenshein, 2008; Bhattacharjee et al., 2016b). Though the hypothesized amino acid germinant receptor has not been identified, the bile acid germinant receptor is the subtilisin-like, pseudoprotease, CspC (Francis et al., 2013). Because this model differs from other models of spore germination, we hypothesized that other organisms whose *csp* locus is similar to that of *C. difficile* may initiate germination through this alternate pathway.

Using NCBI BLAST to search for *cspBAC* loci similar to *C. difficile* yielded a couple of Clostridial species with high genetic similarity, *P. bifermentans* and *P.* *sordellii* (Kevorkian et al., 2016). *P. sordellii* is a virulent organism which cause a range of health issues such as hemorrhagic enteritis in animals, infections due to penetrating trauma (e.g., black tar heroin use), and gynecological procedures in humans (Al-Mashat and Taylor, 1983; Clark, 2003; Kimura et al., 2004; Fischer et al., 2005; Aldape et al., 2006; Aronoff and Ballard, 2009; Ramirez and Abel-Santos, 2010). *P. bifermentans*, rarely, is associated with disease directly, with only 13 cases reported (Hale et al., 2016). Recently, a subspecies, *P. bifermentans* subsp. *malaysia*, was discovered and produces toxins that are active against a host of mosquito genera, especially *Anopheles*, carrier of malarial parasite (Charles et al., 1990). The genetic similarity between *P. bifermentans* and *P. sordellii* is high and, at one point, the two species had been classified as one organism, suggesting that their mechanisms of germination are likely shared (Brooks and Epps, 1959; Maclennan, 1962).

Similar to what is found in *C. difficile*, *P. bifermentans* encodes a translational fusion between *cspB* and *cspA*. Encoded downstream and, likely, part of the same transcriptional unit is *cspC*. Of the three encoded proteins, only CspB is predicted to have catalytic activity (Rawlings and Barrett, 1993; Rawlings et al., 2014). In CspB, the catalytic Asp, His and Ser [and the residues surrounding the triads common for Peptidase S8 family of proteases (subtilisin)] are found between amino acids 96–98, 160–163, and 458–464, respectively (Rawlings et al., 2014). For CspA, only the catalytic Asp is positioned correctly within the surrounding amino acid motif characteristic of S8 family members (amino acids 664–666 of CspBA) (Rawlings et al., 2014). Though the His and Ser residues are present, the surrounding amino acid motifs that are characteristic of the S8 family of peptidases are not in tact [the amino acid motifs surrounding the catalytic His and Ser are one amino acid and three amino acids off, respectively (**Supplementary Figure S2**, highlighted residues)] (Rawlings et al., 2014; Kevorkian et al., 2016). In *P. bifermentans* CspC, the would-be catalytic His and Ser are absent (amino acids 167–170 and 482–487, respectively) but the Asp is present (amino acids 105–108) (Rawlings et al., 2014). Thus, similar to *C. difficile*, only CspB has predicted catalytic activity. Though CspC is a pseudoprotease, CspA may have no catalytic activity or, more likely, may have reduced activity due to the motif for the triad being off by one amino acid for two of the residues.

In the NCBI database, *P. bifermentans* is predicted to encode a *gerA* homolog. GerA belongs to the Ger-type family of germinant receptors. Because we were interested in understanding the mechanism of *P. bifermentans* spore germination, we searched for homologs of the *P. bifermentans* GerA spore germination protein to identify other organisms with mechanisms of spore germination that might resemble *P. bifermentans*. Interestingly, though the *P. bifermentans* gene is annotated as *gerA* in NCBI and UniProt databases, when used as a BLAST query to *B. subtilis*, the most closely related protein identified was not GerA, but rather, SpoVAF. Thus, it is probable that, similar to *C. difficile*, *P. bifermentans* does not encode orthologs of the *ger*-type germinant receptor.

Germination of *P. bifermentans* spores is initiated in response to ARF, though other combinations can stimulate spore germination (i.e., AR and AF) (**Figure 1**). L-alanine is the most common germinant among all, studied, spore-forming bacteria (Bhattacharjee et al., 2016b). Thus, it is not surprising that *P. bifermentans* spores initiate germination in response to L-alanine. Waites and Wyatt (1971) previously characterized the germinants for *P. bifermentans* spores and described lactate and pyruvate as germinants. Since we observe rapid and efficient germination in response to ARF, as measured both by OD change and DPA release (**Figures 1C,D**), we did not test pyruvate or lactate with the amino acid mixture. Because both *C. difficile* and *P. sordellii* encode *csp*-type receptors and both respond to steroid-based compounds (bile acids) as cues for germination, we tested if the presence of these proteins results in a spore that responds to bile acids as germinants (Sorg and Sonenshein, 2008; Liggins et al., 2011). As shown in **Figure 2**, the rate of *P. bifermentans* spore germination, measured by changes in OD_600_ nm, was enhanced by both TA and DCA. However, this effect on germination was not apparent when the release of DPA was analyzed. Potentially, TA and DCA help germinants gain access to the germinant receptors or help cortex degradation. However, the reason for the impact of the bile acids on germination, measured by changes in OD_600_ nm, is unknown. Thus, bile acids do not appear to impact the *P. bifermentans* spore germination and, therefore, the mere presence of the Csp proteins does not indicate that a spore germinates in response to steroid molecules. This is similar to other germinant receptors. Despite homology among the Ger-family of germinant receptors, there are differences in the signals that stimulate germination. For example, the *B. subtilis* GerAA protein is 43% identical (62% similar) to the GerBA protein. These two germinant receptors respond, at least in part, to very different germinants (L-alanine or AGFK, respectively). The *C. difficile* CspBA protein is 59% identical and 75% similar to the *P. bifermentans* CspBA protein (**Supplementary Figure S2**) suggesting that, should the CspBAC proteins from *P. bifermentans* function similarly to that of *C. difficile*, these proteins could respond to different germinants (Kevorkian et al., 2016).

In prior studies, we found that the release of cortex fragments by germinating *C. difficile* spores precedes the release of DPA from the core and that high concentrations of osmolytes (i.e., sorbitol, trehalose, or sucrose) could delay the release of DPA from the core of germinating *C. difficile* spores (Francis et al., 2015; Francis and Sorg, 2016). Similar to what is observed during *C. difficile* spore germination, we found that cortex fragments appear in the germination solution before DPA during *P. bifermentans* spore germination. Moreover, the release of DPA by germinating *P. bifermentans* spores could be delayed by sorbitol in a dose-dependent manner. Because cortex degradation preceded DPA release during *P. bifermentans* spore germination, these results suggest that DPA release by germinating *P. bifermentans* spores occurs in a mechanosensing fashion. Because the rate of DPA release *B. subtilis* was not as affected by high concentrations of osmolyte, mechanosensing is likely not important for DPA release during nutrient-mediated germination; DPA-mediated germination likely would stimulate cortex degradation and thus release of DPA from the core in a mechanosensing fashion (Setlow, 2013; Velasquez et al., 2014).

Prior work done by Kevorkian et al. (2016) revealed that the Peptostreptococcaceae family members conserve a catalytically dead CspC protein but the CspBA proteins vary in their hypothesized protease activities. For example, *C. difficile* encodes a functional CspB protein (F) fused to a non-functional CspA protein (N). This FN arrangement is conserved across all *C. difficile* isolates, but is not universally conserved in all Peptostreptococcaceae. For *P. bifermentans*, the authors found an arrangement of a non-functional CspB but a potentially functional CspA. Based upon our findings here, CspB is likely to be catalytically active (due to the sequencing error in the published genome) and CspA may be inactive or exhibit reduced activity (see above). But, pseudoprotease regulation of spore germination may be a common feature of the Peptostreptococcaceae family. Moreover, in the absence of Ger-family germinant receptors, organisms that encode Csp pseudoproteases may germinate in an “outside-in” mechanism.

In summary, we found that *P. bifermentans* spore germination occurs most similarly to that observed in *C. difficile*. Unfortunately, due to the lack of a genetic system in *P. bifermentans*, we could not directly test the effects of mutations introduced into the *cspBA* or *cspC* coding regions. However, based on the appearance of cortex fragments before DPA during germination of *P. bifermentans* spores and that high osmolyte concentrations can delay the release of DPA, we predict that *P. bifermentans* spore germination proceeds through the same pathway as observed during *C. difficile* spore germination. These findings build upon the hypothesis that Csp-type germinant receptor activation stimulates spore germination though an ‘outside-in’ direction and represents a novel germination pathway involving pseudoproteases.

## Materials and Methods

### Bacterial Strains

Wild type *P. bifermentans* ATCC 19299 was purchased from the American Type Culture Collection (ATCC) and grown in an anaerobic atmosphere (10% H_2_, 5% CO_2_, and 85%N_2_) at 37°C on Difco Reinforced Clostridial Medium agar (RCM) medium, as recommended by ATCC. *B. subtilis* PS533 was grown on Difco sporulation medium (DSM) and LB medium.

### Sporulation

*P. bifermentans* cells were streaked onto pre-reduced Duncan-Strong Sporulation Media (DSSM) agar plates (Duncan and Strong, 1968) under anaerobic conditions at 37°C. The cells were allowed to grow for 3–4 days before harvesting by scraping the growth into sterile water. *B. subtilis* cells were streaked onto DSM agar medium and allowed to grow for 4 days at 30°C, as described previously (Francis et al., 2015), and harvested as described above.

### Spore Purification

*S*pores, vegetative cells, debris, and any agar that contaminated the harvested preparation (*P. bifermentans* and *B. subtilis* grew into the agar surface) were stored overnight at 4°C. Agar was removed from the scraped spores by incubating the suspension at 75°C for 1 h, as described previously (Francis et al., 2015). The resulting suspension was washed 5 times in sterile water and purified on 60% (w/v) sucrose solution as described previously (Bhattacharjee et al., 2016a). Purified spores were again washed 5 times and stored in 1 mL sterile water. Purified spores appeared phase bright and did not contain observable vegetative cells.

### Spore Germination

Purified *P. bifermentans* spores were heated for 30 min at 75°C (heat activation was required for *P. bifermentans* spore germination; **Supplementary Figure S3**) and *B. subtilis* spores at 80°C prior to germination (Keynan et al., 1964; Francis et al., 2015). Spores were added to Falcon clear 96 well plates containing germination buffer (50 mM HEPES pH 7.5, 100 mM NaCl) alone or in buffer supplemented with germinants (50 mM L-alanine, 5 mM L-phenylalanine, 5 mM L-arginine for *P. bifermentans*; 100 mM L-valine for *B. subtilis*). Sorbitol was added where indicated. Germination of the OD_600_ = 0.5–0.7 spore suspension was measured over time at OD_600_ nm in SpectraMax M3 plate reader at 37°C.

The release of DPA from OD_600_ = 0.25–0.3 spores was measured by adding 250 μM terbium chloride (final concentration) to germination buffer (above) with or without added germinants and/or osmolyte, as described previously (Bhattacharjee et al., 2016a). Terbium (Tb^3+^) fluorescence was monitored using a SpectraMax M3 plate reader with excitation at 270 nm and emission at 545 nm with a 420 nm cut-off as previously described (Bhattacharjee et al., 2016a).

### Cortex Hydrolysis

Cortex hydrolysis assay was performed for *P. bifermentans* spores, as previously published (Francis et al., 2015). Spores were suspended in germination buffer at OD_600_ ∼ 3. However, the germinant concentrations were increased to 100 mM each of L-alanine, L-phenylalanine, and L-arginine to increase germination of the spores.

### Statistical Analyses

Data points represent the average from three, independent experiments and error bars represent the standard deviation from the mean. Statistical significance was determined using a two-way ANOVA with Sidak’s multiple comparisons test.

## Author Contributions

DB performed the experiments. DB and JS analyzed the data and wrote the manuscript.

## Conflict of Interest Statement

The authors declare that the research was conducted in the absence of any commercial or financial relationships that could be construed as a potential conflict of interest.
