# Supplementary material for: Conservation of the “Outside-in” Germination Pathway in Paraclostridium bifermentans
Source: Front Microbiol. 2018 Oct 17;9:2487. doi: 10.3389/fmicb.2018.02487 (PMC6199464; doi:10.3389/fmicb.2018.02487)
Supplement: Supplementary file 1 [file Data_Sheet_1.PDF]

Supplemental Material

Figure Legend

Figure S1. Frameshift mutation in *P. bifermentans cspBA* in the deposited NCBI sequence. The *P. bifermentans csp* locus was identified from the unannotated, unassembled *P. bifermentans* genomic sequence (Accession Number: NZ_AVNB00000000.1) by comparison to *C. difficile* R20291 *cspBA* and *cspC* genes (Accession Number: FN545816.1) The *P. bifermentans cspBA* gene was sequenced using the primers 5' – GGAAGCTAGTTTTTACACCG – 3' and 5' – GGTAGTGTTAATACCCCCTC – 3'. This sequencing led to the identification of a -1 frameshift in the deposited NCBI sequence.

Figure S2. Alignment of the *C. difficile* and *P. bifermentans* CspBA, CspC and SleC proteins. The *C. difficile* and *P. bifermentans* CspBA, CspC and SleC sequences were aligned in DNASTAR MegAlign using the Clustal W method. The residues at catalytic sites are highlighted in red, the conserved sequences surrounding the catalytic residues are highlighted in black, and deviation from the consensus sequence for S8 proteases are highlighted in green.

Figure S3: Germination of non-heat-treated *P. bifermentans* spores. *P. bifermentans* spores were purified as described in the materials and methods. The un-heat-treated spores were suspended in buffer alone (□) or in buffer supplemented with 50mM Ala, 5mM Phe and 5 mM Arg (■).

Figure S1

**cspBA (NCBI)**	...TTTACTTTATAGAAAAAAGAAAAAAAAAGTTAAA...
**cspBA (sequenced)**	...TTTACTTTATAGAAAAAAGAAAAAAAAA**A**GTTAAA...

Figure S2

cspBA

```
P. bifermentans    VFFINYEVIVKYNGDISEIEKDLGVSVEKLGYNYAIIITDSAEKIDLLLNYPQIEYLEKP
C. difficile       VIIINYELIVKYNGDILRLEEELGVSVEILNSSYAIITSSNEEDVNTLLTYPEIEFIEKP
                   *::****:******** .:*::****** *. .**** :.. *.:: **.**:**::***

P. bifermentans    FILTTQDIQSFSRTGITRFKNTNRLTGKGTIIGIIDSGIDYNIDLFKDSNGNSKILYYWD
C. difficile       FILQTQDVQSFSSTGITGFKNRTGLTGKGTIIGIIDSGIDYTLPVFRDSDGRSKILYYWD
                   *** ***:**** **** *** . ****************.: :*:**:*.********

P. bifermentans    QSIKGKPPEGFQYGSLYTNEDINKAIKRELNIPISPTSTHGTHVAGIACQIAEEANLIVV
C. difficile       QSIQGNPPEGFREGTLYTNEDINNAIDGSMYIPISTTSLHGTHVAGICATIASDARIIVV
                   ***:*:*****: *:********:**. .: ****.** ********.. **.:*.:***

P. bifermentans    RVGSVVTDVFSKSTEFMRAIKFILDKALELKMPAAINISYGSNEGSHRGLSLFEQYIDDM
C. difficile       RVGNIQTDIFSRSTEFMRAIKFILDRALELRMPVTLNISYGSNEGSHRGTSLFEQYIDDM
                   ***.: **:**:*************:****:**.::************* *********

P. bifermentans    SAFWKNNIVVAAGNNGDKDGHKSIKLDK-ETTEVEFVVGENEKILNINIWPEFIDDFSVY
C. difficile       CLFWKNNIVVAAGNNADKGGHKRIRLQNNITEEVEFIVGEGERILNINIWPDFVDDFSVH
                   . *************.**.*** *:*::  * ****:***.*:*******:*:*****:

P. bifermentans    IVNPSNIKSQEISLTSGEIKNVLGGTRVKGYFYPITPYSLSRRISVQLTSPTFINPGIWK
C. difficile       LVNPSNNQTQAISLTSGEIRNTLGETRITGYFYPIAPYSLTRRVTLQLSSNTQITPGLWK
                   :***** ::* ********:*.** **:.******:****:**::::**:* * *.**:**

P. bifermentans    LVFTPINIVMGDISIYLPTSEGISKDTRFLEANKNLTVTVPGTANKVITVGSFNSTTDTV
C. difficile       IVFEPIDIVTGNVNIYLPTSEGLNRNTRFLIPTQELTVTVPGTASRVVTVGSFNSRTDIV
                   :** **:** *::.********:.::****  ..::*********.:*:******* ** *

P. bifermentans    SIFSGEGDIDQNVYKPDLLAPGENILSVLPGGSIGALTGTSMATPHVTGVVSLLMQWGIV
C. difficile       SIFSGEGDTQLGVFKPDLLAPGEDIISFLPGGTSGALTGTSMATPHVTGVCSLFMEWGIV
                   ******** : .*:*********:*:*.****: *************** **:*:****

P. bifermentans    DKNDLFLYSQKIKAFLLKEAKRNSTYTYPNNPMGFGFLDLTEVRLDNISNLNKEYDLLYR
C. difficile       NGNDLFLYSQKLRALLLKGARRLSNQSYPNNSSGFGFLNLSDIDLYTLSSINQDLETEDI
                   : *********::*:*** *:* *. :****. *****:*::: * .:*.:*:: :

P. bifermentans    KRKKKVKELSRLDLPLDLAFRYHVEHG----------------------------PNFEE
C. difficile       GYRSINKSFKDEENRYKFIDGYNIQIHNDLENEIYISKNASRQSGILSGIDVVHTPEFEE
                     :.  *.:.  :   .:   *:::                               *:***

P. bifermentans    ELKAIGLDYTYYEISDTEGVLTLPIKDPKRYNKLLKIEGFKFIASSLVMNQLGTISRDTS
C. difficile       ELAGLGMSQNFFKISDSLGVLSINNTDYSSIQRVLQLPSIIRTVSTTKMTLLGEINRGTF
                   **  .:*:.  .:::***: ***::   .*  .   :::*:: .:    .*:   *. ** *.*.*

P. bifermentans    NGVVAKEEIGANFLQNNLNIPITGSGVLVAVLDSGIDYLHEDFIYPDKTSKIAYIWDQTE
C. difficile       GGVVATEEMGVNFFKNNPNINITGRGTLISIADTGIDYLHPDFIYPDGTSKIVYLWDQTK
                   .****.**:*.**::** ** *** *.*::: *:****** ****** ****.*:****:

P. bifermentans    DGNPPKGYKIGTEYSREDINKAIQANDNNLTKDETGHGTMISGICSGLGNLNKQYSGVAP
C. difficile       EGTPPDGFYIGTEYTREDINRAIAENDPSLSQDEVGQGTMLSGICSGLGNVNSEYAGIAE
                   :*.**.*: *****:*****:**  ** .*::**.*:***:*********:*.:*:*:*

P. bifermentans    DSELIIVKLKKIEGNYDSTFLKAGVMYAYEKSIELNKPIVINLSLGSNNLIGAKENTLRK
C. difficile       DSELIIIKLGKIDGFYNSAMLFAASQYAYKKAFELRRPLVINMSLGTSSLAGLTNRSNSE
                   ******:** **:* *:*::* *.  ***:*::**.:*:***:***:..* * .:.:  :

P. bifermentans    EPHFARGVCIVSAVGNEGNTQTHSMGKVNFVGQQKDIEIEIFEDESLLEINIWVNKPDKI
C. difficile       KAFFTRGLCITAGAGNEGNTQTHTSGIIPHVGGSVEVELELNEDEEELSLELWLNRPDKA
                   :..*:**:**.:.:.*********: * : .** . ::*:*: ***. *.:::*:*:***
```

```
P. bifermentans   SAAVIAPSGEQSKFITVSSYNEVSGLFDLESTWYVITYVYPTEYSGQEQITVLLKNATKG
C. difficile      DVIIVSPTGEESKSVGISNYNKVTGLFDLEGTEYSITYIYPTTFSGQQFTNVTLKNAKRG
                  .. :::*:**:** : :*.**:*:******.* * ***:*** :***:  .* ****.:*

P. bifermentans   IWKIRLRGEYITNGIYNAYLPNRAIINPGTKFRDSTSAYTINYPATYRDVISAGAYNLVE
C. difficile      VWKIRLVGVYIITGRYNLYLPNRELLKSGTRFREVDPFYTINYPAIQDDLITVGAYNTIN
                  :***** * ** .* ** ***** :::.**:**:  . *******   *:*:.**** ::

P. bifermentans   NSIWPPSSRGPTINGLLRPDIVAPGVNIISTYPGNTYATLTGTSASGAYLAGSVALYLQY
C. difficile      GSLWQSSSRGPTIEDRLKPDIVAPGVNIIAAYPGNTYATITGTAAASAHAAGAAAMYFQY
                  .*:* .*******:. *:***********::********:***:*:.*: **:.*:*:**

P. bifermentans   TLVDNYYPLKGFTNIIRTYIRAGAKRNSEIVYPNDIYGYGILDIRGAFDQLK
C. difficile      TFVDGRYPNQAYVQKIKTFMQAGARKDSNTVYPNTNSGYGLLDVRGMFDVLR
                  *:**. ** :.:.: *:*:::***:::*: ****   ***:**:** ** *:
```

cspC

```
P. bifermentans     LEKSYLIIYKGDIASDLKKAGIEKYMILNPSLTVIYVPQNFREETLNRIPSITWWQSSIP
C. difficile        MEKSYCIIYQGDIESALQENGINRYMVLNSQLAVIYVPVDFDETILNNIIQVAWWEESEP
                    :**** ***:*** * *:: **::**:**..*:***** :* *  **.* .::**:.* *

P. bifermentans     MSSLIEITNNLDEGVSVSDAASTDYIYKNPYIQSTGRNVLIAIIDSGIDYLHPDFMENN-
C. difficile        MSSLIEITNNVNNGETITTAAETDYIYENPYNDITGRGILLAVIDSGIDYLHPDFINDDG
                    **********:::* ::: **.*****:*** : ***.:*:*:************:::

P. bifermentans     KTKIISIWDQESEKKNPPDGLIFGSEFTSEDINKAIEENDKTLSEDSIGTGTTAAGIAAG
C. difficile        TSKVLYLWDQEANTNPPPEGFIFGSEFTRSQLNIAINRNDGSLSQDNIGTGTLVSGILAG
                    .:*:: :****::.: **:*:******* .::* **:.** :**:*.***** .:** **

P. bifermentans     RGNLNSQYKGVAIDSKLVVVKLREYKDTYKKGKINYQGSDFLASIRYVLDVAKKENKNMI
C. difficile        NGRINSQYRGITTESDLIVVKLKSYTDTYYAGRINYSVSDFLAAITYVTNIARTENKPLI
                    .*.:****:*:: :*.*:****:.*.***  *:***. *****:* ** ::*:.*** :*

P. bifermentans     INLTVGLISKSIVESTMLSTFNELSQPGNIVVSGAGNEGNTDIHYRGNIKNKETVDDIII
C. difficile        INLTIGVKSSAVATTSILDTFNILSSAGVVVVSGAGNQGNTDIHYSGRFSSVGEVQDVII
                    ****:*: *.::. :::*.*** **..* :*******:******* *.:..   *:*:**

P. bifermentans     QVGEQTNLDIKLVVNGPDKIGAMIISPAGEMSYKIMYSPDYYVYKGKFNLESTPYEMRLS
C. difficile        QDGDDYALDITLNTNGPDKVGAQIISPSGEVSHDIRYSPDFYIYRGKFNLENTTYAMRFI
                    * *::  ***.* .*****:** ****:**:*:.* ****:*:*:******.*.* **:

P. bifermentans     YPWLESGNEELTISLYDIKPGIWTLRLIPEFIIEGNYDVYLPNKNIISEEARFSDPASEA
C. difficile        YPYITSGKENLEIRLRDIKPGVWILRLTSELIISGEYDIYLPNKNLIAPDTRFLDPDSVA
                    **:: **:*:* * * *****:* *** .*:**.*:**:******:*: ::** ** * *

P. bifermentans     TISMYAASENVITIGAYNDKTDSIWIGSSKGPVNLDLIKPDIVAPGVDIISTYINSSYNT
C. difficile        TITMYAASDDVITVGTFNNKTDSMWIGSSKGPIRGRGIKPDIVASGVDIISTYKNGTYNT
                    **:*****::***:*::*:****:********:.   *******.******** *.:***

P. bifermentans     SIGTGVSSSIVSGVLAIILEYITSEYEFAEELLSVQPLKTYLMLGATKKDIYIYPNITQG
C. difficile        GTGTGVSSSIVTGVLALLMEYLEKQDNVPRLSLFTQVLKTYLILGATKLEIYTYPNVSQG
                    . *********:****:::**: .: :...  * .* *****:***** :** ***::**

P. bifermentans     YGILNLKNTIVEIAKNFE
C. difficile        YGILNLKNTIQQIANTL-
                    ********** :**:.:
```

sleC

```
P. bifermentans      KYIFSFPAFFSLIKFSLPLLKCTSNIIILHRVPFYLLFFKIFTFFNLFYFHHKINLILQM
C. difficile        ------------------------------------------------------------

P. bifermentans      VHFKYFIHIYKLIDYLGGIFLYKGLLTIKVTDEITNFPIEGVSINICAMPKEGSTKSKYI
C. difficile        --------------------MQDGFLTVSIIDATNNRPIQNAVVNIYSMSN-GSQSSSTL
                                        : .*:**:.: *  .* **:.. :** :*.: ** .*. :

P. bifermentans      YKNLITNSSGMVKKVSLDAPNFIYSQVPNSPRAYSTYILTISKDGYQSVVIQGVQILPLV
C. difficile        YQNLRSNESGQVTGLVLPAPDVDYSLQPSDVRPYSQYIVEAIADGYETVVIEGTQLLATI
                    *:** :*.** *. : * **:. **  *.. *.** **:    ***::***:*.*:*. :

P. bifermentans      EAIQNISLSKIS----AFTTTNKKIYKIGDNVLYGNYQPKILEDDLKKVP-----YVLPN
C. difficile        VARQGVPMSPRTRSKRSFSRQSELIFDIGEHTLYGTYPPKIPESNLKPLPPPTGFVVLDN
                     * *.:.:*  :    :*:  .: *:.**::.***.* *** *.:** :*      ** *

P. bifermentans      VVVPEYIIVHDGMPSDKNAPNYWIPFRDYIKNVASSEIYATWPTETIYANVVAIVSFTLN
C. difficile        PVVPEFIVVHDGLPEDSSAPNYWIPFKEYIKNIASSEIYSTWPEQTIYANVIAIISFTLN
                     ****:*:****:*.*..********::****:******:*** :******:**:*****

P. bifermentans      RVYTEWYRNMGYDFTITSTTAYDHKFIYNRNIFDTISVVVDNIFNVYIQRPKGNPQPLLA
C. difficile        RVFTEWYRNKGYNFTITSTTAYDHKFINNRNLFEPINVVVDAIFNTFIKRPPTSRQPLLA
                    **:****** **:************** ***:*:.*.**** ***.:*:**  . *****

P. bifermentans      QYCDGIQTQCPGKMTQWGSKYLGDQGYKFDEILRYYYGQDIGLQGTDMIKGVPSSFPGYT
C. difficile        QYCDGQKSQCPDQMTQWGSKDLGDQGYDYESILRYFYGDEIVFERAPIVSGVPVSFPGTT
                    ***** ::***.:******* ******.::.****:**::* :: : ::.*** **** *

P. bifermentans      LTLWSTGEPVRTIQNQLNAIANAYPALPKVDVDGIYGPKTQESVRKFQEIFRMTQSGNVD
C. difficile        LQVGSSGQYVRTIQNQLNAISNSYPAVPKVIEDGIYGADTENAVKIFQGIFGLPQSGVVD
                    * : *:*: ***********:*:***:***  *****..*:::*: ** ** :.*** **

P. bifermentans      FATWYAISKIYVAVTKIAEFEI--
C. difficile        FKTWYEISRVYYVATTRIASLNPLI
                    * *** **::***.*:**.::
```

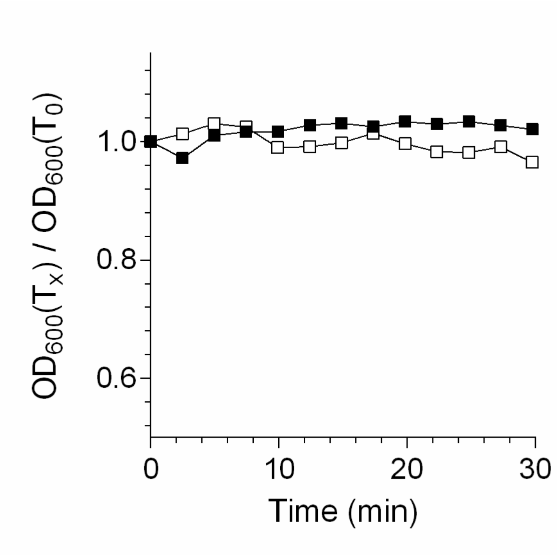

Figure S3
